# Diverse Regulation but Conserved Function: SOX9 in Vertebrate Sex Determination

**DOI:** 10.3390/genes12040486

**Published:** 2021-03-26

**Authors:** Brittany Vining, Zhenhua Ming, Stefan Bagheri-Fam, Vincent Harley

**Affiliations:** 1 Sex Development Laboratory, Hudson Institute of Medical Research, Melbourne, VIC 3168, Australia; Brittany.vining@monash.edu (B.V.); Zhenhua.Ming@monash.edu (Z.M.); stefan.bagherifam@gmail.com (S.B.-F.); 2 Department of Molecular and Translational Science, Monash University, Melbourne, VIC 3800, Australia

**Keywords:** SOX9, transcription factor, evolution, transdifferentiation, sex determination

## Abstract

Sex determination occurs early during embryogenesis among vertebrates. It involves the differentiation of the bipotential gonad to ovaries or testes by a fascinating diversity of molecular switches. In most mammals, the switch is *SRY* (sex determining region Y); in other vertebrates it could be one of a variety of genes including *Dmrt1* or *dmy.* Downstream of the switch gene, *SOX9* upregulation is a central event in testes development, controlled by gonad-specific enhancers across the 2 Mb *SOX9* locus. *SOX9* is a ‘hub’ gene of gonadal development, regulated positively in males and negatively in females. Despite this diversity, SOX9 protein sequence and function among vertebrates remains highly conserved. This article explores the cellular, morphological, and genetic mechanisms initiated by *SOX9* for male gonad differentiation.

## 1. Introduction

Sexual development and the mechanisms underlying sex determination have been a key interest of developmental and evolutionary biologists. The embryonic gonad has been revered as the ideal model for the investigation of organogenesis, as the choice between male and female cell lineages from the bipotential gonad provides valuable information about the molecular regulation of cell fate and pattern formation. Vertebrate sex determination during embryogenesis is usually controlled by a genetic “switch” in which the development of the testes or ovaries arises from the bipotential gonads. Using model systems such a mealworms [1], *Drosophila melanogaster* [2], rabbits [3], *Caenorhabditis elegans* [4] and *Mus musculus*, the understanding of sex determination and testicular development has progressed. Reptiles [5,6,7], avian species [8,9], monotremes [10,11] and fish [12,13] display various genetic and environmental triggers of sex determination and gonad differentiation. Yet, there is the possibility for cellular transdifferentiation such that the sexual fate of post-natal gonadal cells is not permanent [14,15]. *SOX9* (*SRY*-box 9) has a conserved role in vertebrate gonadal development, underpinned by a variety of switch mechanisms that control its expression for sex determination.

The apparent conserved role of *SOX9* in male sex determination and the highly conserved protein sequence suggests a conserved function for SOX9 as a transcriptional activator of male-promoting target genes. However, the sex determination switch that controls *SOX9* expression is not conserved across vertebrate species, despite the importance, and conservation, of sexually dimorphic features [16]. Across taxa, crocodilians, many turtles and fish rely on environmental cues such as temperature for sex determination, indicating there is no genetic predisposition for a temperature-sensitive species to develop as female or male until the thermosensitive period of development [5,6,17,18]. Male humans, mice, and most mammals have heteromorphic XY chromosomes in which the Y chromosome harbours the male-dominant sex-determining *SRY/Sry* gene (Sex determining region of Y chromosome) [19,20,21], whereas females carry XX chromosomes. In avian sex determination, females are heterogametic ZW, and males are homogametic ZZ, with the avian Z chromosome harbouring the dosage-dependent sex determining gene *DMRT1* (Doublesex and mab-3 related transcription factor 1) [8]. Amphibians can have either or both XY/XX or ZW/WW sex determining mechanisms [22] and fish have quite diverse chromosomal sex determining mechanisms [23]—for example, the medaka has an XY system with *dmrt1 (dmy)* as the sex determining gene [13]. The platypus presents a peculiar sex determination system: males have five X and five Y chromosomes, lacking an *Sry* gene; females have five pairs of X chromosomes, and the platypus still harbours an autosomal *Sox9* gene [10,24].

Sex determination can be considered a contest between pro-testes and pro-ovarian genes; for example in most mammals, the presence of the Y chromosome tips the balance in favour of the male cell fate [25]. At the genetic level, there is molecular antagonism among the positive and negative regulators (transcription factors) and regulatory regions (enhancers and repressors) acting on *SOX9*. This article will review cellular and morphological changes unique to early formation of the male gonads, with *SOX9* function being conserved among species yet regulated via different mechanisms, and how this information fits into the comprehensive network of gene regulation during and after sex determination.

## 2. Unique Cellular and Morphological Changes during Male Gonad Development

Sex determination occurs when bipotential gonads differentiate into testes or ovaries, usually due to the karyotype of the developing fetus. Sexually dimorphic species, in which there are two distinct sexes, rely on sexual reproduction to produce offspring with genetic variation, such as the case with mammals, birds, and reptiles. While vertebrates vary in the processes of sex determination and chromosomal features, they can have relatively similar structural anatomy [26].

**Figure 1:**
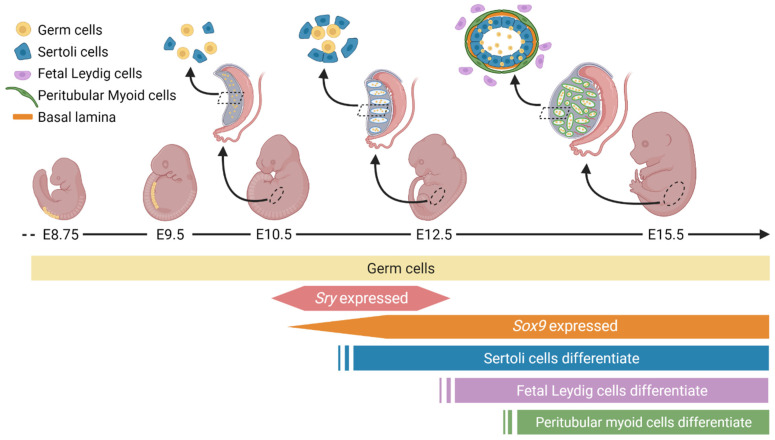
Morphological changes of the differentiating mouse testis: Between embryonic day (E)8.75 and E9.5, germ cells migrate dorsally towards the developing genital ridge. The testis-determining factor SRY activates *Sox9* expression to drive Sertoli cell (blue) differentiation from E10.5. Proliferating Sertoli cells begin to compartmentalize to form testis cords around E12.5. Following Sertoli cell proliferation, fetal Leydig cells (purple) and peritubular myoid cells (green) differentiate. By E15.5, the majority of testis cell types have differentiated, and a distinct mouse testis has formed with typical testis cord structure and interstitial space. The Sertoli cells and peritubular myoid cells secrete various extracellular matrix (ECM) proteins to form the basal lamina, which surrounds the testis cords and maintains their structural integrity. The testis cords are composed of mitotically arrested germ cells enclosed by Sertoli cells, with an outer layer of peritubular myoid cells and extracellular matrix. The interstitial space comprises steroidogenic fetal Leydig cells, mesenchyme, and a prominent blood vasculature.

The genital ridge is an undifferentiated region from which testes and ovaries form, comprised of a narrow band of proliferating cells, appearing in mice around embryonic day (E)10.5 [27,28] and in humans around gestational week 4 [29]. The primordial germ cells migrate to colonise the thickening genital ridge (Figure 1) [27,30,31] to associate with undifferentiated somatic cells [32,33]. After E10.5 in mice, sex differentiation diverges, whereby the bipotential gonad commits to either the testicular or ovarian pathway [33]. Supporting cells commit to develop as either Sertoli or granulosa cells, steroidogenic cells commit to either Leydig or theca cells, and germ cells prepare for spermatogenesis or oogenesis later.

Sertoli cell differentiation in humans and mice depends on the expression of a unique gene residing on the Y chromosome, and only present in males, known as the *SRY/Sry* gene [19,20]. SRY in turn upregulates the transcription of the highly conserved gene *SOX9/Sox9*. Sertoli cell proliferation in mice from E11.25–E13.5 causes the gonadal width to double every 24 h [32] and consequently Sertoli cells aggregate to form the presumptive seminiferous tubules [34] (Figure 1). Exposure of mouse XY gonads to inhibitors of proliferation during the critical window of E10.8–11.2 results in a failure of cord formation and reduced expression of male-specific genes [34]. This critical window also aligns with the onset of SRY and SOX9 expression and the onset of early male sex determination. Following differentiation, Sertoli cells direct the compartmentalisation of the testis cords and the interstitial space around E12.5 [32]. Testis cords encapsulate the germ cells, and Sertoli cells work in conjunction with peritubular myoid cells to deposit a basal lamina (Figure 1). This deposition is critical to provide structural integrity to the testis cords and separate them from the interstitial space to provide the right conditions for spermatogenesis later.

In summary, Sertoli cells are a key component of testis differentiation, driven by the expression of SRY and SOX9. It is remarkable that three unique cells lineages in the bipotential fetal gonad (supporting cells, steroidogenic cells, and germ cells) have the capacity to differentiate into testicular or ovarian lineages. This fate commitment, once thought to be permanent, requires constant reinforcement to prevent transdifferentiation [14,15]. Understanding the molecular mechanisms that drive gonadal differentiation can facilitate the understanding of the genes and pathways related to disorders of sex development (DSD) and provide understanding of differentiation and development of other organs during embryogenesis.

## 3. SRY and DMRT1: Key Switches in Sex Determination

Historically, the sex determining region of the Y chromosome in humans and mice was given the acronyms *TDF* (Testis-determining factor) and *Tdy* (testis-determining region on the Y chromosome), respectively [35]. Now, the gene determining male sex has been identified and named *SRY* in humans and *Sry* in mice [19,20]. This gene has been previously well reviewed [36,37,38,39,40], and will not be comprehensively covered in this article. To fully understand the development of the testis it is important to briefly discuss *SRY/Sry*—the gene essential for male sex determination.

*SRY/Sry* is expressed exclusively and transiently in the supporting cells of the genital ridge to direct cells to develop as Sertoli cells [41,42]. In mice, *Sry* gene expression begins at E10.5, reaching peak expression at E11.5 then declining rapidly to an undetectable level by E12.5 [43]; in humans, *SRY* gene expression is initiated around day 40 post conception, plateauing around day 48 [42]. The expression of the mouse SRY protein first appears centrally in the developing gonad, further moving towards the posterior and anterior poles [44]. The resulting Sertoli cells continually express SOX9 during testicular development. SOX9 acts as a part of a cascade, initiating a positive feedback loops for SOX9 expression, and upregulating expression of anti-Müllerian hormone (AMH), Desert Hedgehog (DHH), Peptidyl arginine deaminase 2 (PADI2), and Prostaglandin D2 synthase (PTGDS) [45,46]. Both SRY and SOX9 contain a sequence-specific DNA HMG-box capable of regulating transcription. Interestingly, while it has been shown that *Sox9/SOX9* is regulated by SRY expression in mice [47] and humans [48], it remains less clear how regulation of *Sry/SRY* occurs [40,49].

*SRY* is not the key sex determining switch in all species as it is only present in mammals. As previously mentioned, *DMRT1* plays an integral role in avian sex determination in a dosage-dependent manner [8]. Expression of *Dmrt1* in the mouse gonad is not observed in a testis-specific manner until E12.5, as expression is observed in somatic cells and germ cells of both sexes until this point [50]. The Japanese rice fish *Oryzias latipes* (Medaka) has an XY system with *dmy* as the sex determining gene [13,51], with *dmy* considered to have appeared via gene duplication of *DMRT1* [52]. Further review of DNA-binding DM domains and the role of *Dmrt1* in many vertebrates has been previously reviewed [53] and will not be covered comprehensively in this review.

## 4. Identification of Testis-Specific Enhancers of *SOX9/Sox9*

Within the unusually large topologically associating domain (TAD) spanning 68.67 to 70.45 Mb on Chromosome 17 in humans (17q24.3), *SOX9* is the only protein coding gene [54,55,56], and tissue and temporal-specific regulation is achieved through complex mechanisms [55]. The specific mechanism by which SRY activates *SOX9/Sox9* has only begun to be elucidated, with previously poor understanding in humans and partial understanding in mice. *SOX9* tissue specific expression is driven by long-range regulatory elements, such as enhancers, within the 2 Mb region upstream of the TSS [48]. Initially, it was proposed that SRY and Steroidogenic factor 1 (SF1, encoded by *NR5A1*) act synergistically in mice to activate a *Sox9* enhancer known as the Testis Specific Enhancer of *Sox9* core (*Tesco*) [47,48,57,58]. This testis-specific enhancer was discovered starting with a bacterial artificial chromosome harbouring a 120 kb genomic fragment with regulatory regions up and downstream of the *Sox9* transcription start site (TSS), and in which the *Sox9* gene was replaced by a *lac-Z* reporter gene [58]. Within a 3.2 kb genomic fragment (TES), a 1.4 kb enhancer (TESCO) mirrored endogenous *Sox9* expression, including onset at E10.5, increased expression at E11.5, and expression restricted to the testis only by E12.5 [58]. After SOX9 expression is initiated, SOX9 creates a positive feedback loop in which it auto-regulates its own transcription via TESCO [58], and *Sox9* expression is maintained via the action of Fibroblast growth factor 9 (FGF9)-fibroblast growth factor receptor 2 (FGFR2) and prostaglandin D2 synthase (PTGDS)-prostaglandin D2 (PGD2) positive feedback loops [59]. However, although deletion of TESCO in mice results in reduced *Sox9* expression in the testis, it is insufficient to cause sex reversal [47]. This implied that *Tesco* is not the sole enhancer required for *Sox9* expression in mice. Human equivalents of mouse enhancers are not always active in mice [60]; the human TES sequence fails to direct testes-specific expression in transgenic mice [60] and no mutations in TESCO have been identified in DSD patients [61].

Within TESCO, an evolutionary conserved region (ECR) of 180 bp exists in mammals, reptiles, birds and amphibians [62]. Within this ECR, highly conserved modules indicate predicted regulatory roles for SOX, DMRT and GATA proteins; this conserved sequence supports the notion that vertebrates might share common aspects of *Sox9* transcriptional regulation despite the diversity of sex determination switches [62].

**Figure 2:**
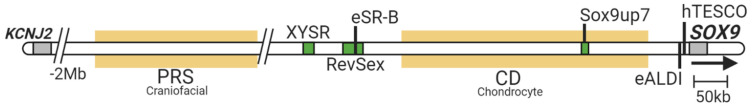
*SOX9* upstream regulatory region towards *KCNJ2* gene. Green regions represent the enhancers that control *SOX9* expression within the testes. Yellow boxes behind the structure indicate regions commonly associated with PRS (Pierre Robin Syndrome) and craniofacial formation, or CMPD (Campomelic Dysplasia) and chondrogenesis. Enhancers eALDI and hTESCO are calculated from Croft et al., [48]. *Sox9up7* is identified from bioinformatic analysis by Ohnesorg et al., 2016 [66].

Through understanding the varied biological mechanisms causing disorders of sex development, the understanding of *SOX9* gene regulation has subsequently expanded, which has led to the identification of several *SOX9/Sox9* testis-specific enhancers. This further highlights *SOX9* as a ‘hub’ gene of gonadal development. XYSR is a regulatory region approximately 500 kb upstream of the *SOX9* TSS, in which 46,XY sex reversal occurs with deletion of the region [63,64,65] (Figure 2). This region was narrowed to 5.2 kb and hypothesised to include a core gonadal enhancer for *SOX9* involved in 46,XY and 46,XX disorders of sex development (DSD) [48].

RevSex (Reversal of Sex), a 24 kb sex determining region 517 kb upstream of *SOX9* was identified through its duplication in patients with isolated DSDs [48,64]. The region was further explored to identify a putative sex reversal enhancer (eSR-B) within the RevSex region. Enhancer ability of eSR-B in a luciferase assay was repressed by the pro-ovarian transcription factor FOXL2 (Forkhead Box L2), despite the stimulatory effect of SOX9 in the same assay [48]. Interestingly, CRISPR/Cas9 deletion of the mouse eSR-B region showed no obvious gonadal or sex reversal phenotype at either embryonic or adult stages, and no significant changes in *Sox9*, *Wnt4*, *Foxl2* or *Amh* mRNA expression levels were observed [48], implying that this particular enhancer may be human specific. An additional SRY-responsive enhancer eALDI (Alternate Long-Distance Initiator) identified in humans shows high resemblance to the functional characteristics of mouse TESCO: it is strongly activated by SOX9+SF1, and deletion of the corresponding region in mice demonstrated that this enhancer, like TESCO, is important for *Sox9* expression levels yet not crucial for male gonad differentiation [48].

More recently, a novel gonadal regulatory element upstream of murine *Sox9* has been identified and named enhancer 13 (Enh13) [48,67]. The 25.7 kb sequence in mice contains an orthologous enhancer to eSR-A of human *SOX9* [48], and homozygous deletion of Enh13 lead to complete XY sex reversal in mice. Most importantly, the conserved region between mice and humans indicates that Enh13 may also have a critical role in human *SOX9* expression and gonadal differentiation. In light of the new regions identified, such as Enh13 and eSR-A, more work is required to understand the role of antagonism and synergistic regulation through these enhancers.

## 5. Vertebrate SOX9 Proteins

*SOX9*, the direct target of SRY, is important during embryogenesis for cellular differentiation of many organs and tissues [68,69,70,71,72]. SOX9 protein is expressed in a variety of tissues, with key functions in cartilage, testis, heart, glial cells, inner ear and neural crest development [56,70,73]. *SOX9* is a member of the SRY-associated HMG-box (SOX) family of transcription factors, specifically subgroup SOXE, that can act upon other genes to regulate or modulate expression for cellular differentiation in a tissue specific manner. SOX-family proteins are defined by their 70 amino acid high mobility group (HMG)-type domain which is at least 50% identical to that of SRY. HMG domains of SRY/SOX proteins are evolutionarily conserved and preferentially bind double-stranded DNA with the AACAAT motif [74]. Upon binding, a DNA bend is induced [75] hence SRY/SOX are considered architectural transcription factors. The molecular basis for DNA recognition and DNA target sites identification of SOX proteins has been recently reviewed elsewhere [76]. Analysis of DNA-binding specificity of SOX9 in vitro by SELEX assay showed that the optimal SOX9 binding sequence, AGAACAATGG, includes a core DNA-binding element AACAAT, flanked by 5′ AG and 3′ GG nucleotides [77].

**Figure 3:**
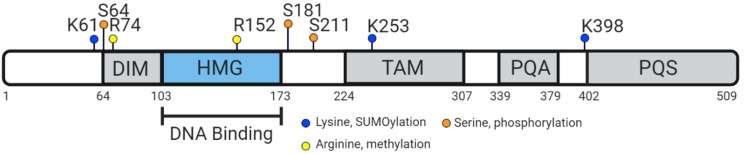
Human protein structure: including the dimerization domain (DIM), DNA-binding HMG-box, transactivating domain in the middle of the protein (TAM), the proline, glutamine, and alanine rich region (PQA) and the proline, glutamine, and serine rich region (PQS), both required for transactivation. Adapted from Symon & Harley, 2017 [60].

The HMG-box contains nuclear localisation sequences (NLS) that bind to calmodulin [78] and importin-β [79]. Defects in the calmodulin-binding NLS can prevent nuclear transportation of SRY, leading to XY sex reversal and ectopic expression of ovarian markers, as the repressed transportation of SRY means it cannot act as a transcriptional regulator of *SOX9* [80,81]. The human SOX9 protein is 509 amino acids with several distinct domains: the defining high-mobility group box, a dimerization domain, a predicted transactivating domain in the middle (TAM), a weak transactivation domain rich in prolines/glutamines/alanines (PQA) and a strong transactivation domain rich in prolines/glutamines/serines (PQS, also referred to as the TAC, or transactivating domain at the C-terminus) [60] (Figure 3). The unique TAC may enhance and mediate transactivation activity in specific contexts through the PQA-rich domain [82,83,84]. SOX9 proteins can homodimerize or heterodimerize with other SOXE proteins via the DIM-HMG interactions (Figure 3) to cooperatively regulate their target genes [85]. Additionally, partner factors cooperate with SOX9 to change genome engagement and target gene expression. For example, in the testis, SOX9 and SF1 recruit each other to the testis-specific enhancers of *SOX9/Sox9* to maintain SOX9 protein expression [48,58]. During chondrogenesis, SOX9 together with SOX5 and SOX6 induce chondrocyte-specific gene expression [86]. In addition, SOX9 functions as a pioneer factor in hair follicle stem cells, capable of binding condensed chromatin, to promote and maintain cell fate [87].

SOX9 is also subject to post-translational modifications: phosphorylation and acetylation to modify nuclear import, and ubiquitination and SUMOylation (small ubiquitin-like modifier) for rate of degradation [88]. Post-translational modifications of SOX9 are observed in many species, such as phosphorylation of Ser64 and Ser181 in mouse, chicken and human SOX9 orthologues [89]. Phosphorylation of SOX9 by protein kinase A (PKA) in response to BMP/TGF-β signaling occurs at Ser64 and Ser181 for chondrocyte differentiation, and SUMOylation through lysine, analogous to ubiquitination, usually occurs to regulate transcriptional repression [89]. The regions in which post-translational modifications for chondrocyte differentiation occur are also within the highly conserved sequence near the N-terminus of the orthologs—further highlighting the notion that in many species, the role of SOX9 shows higher conservation during chondrogenesis compared to sex determination. CARM1 methylation of SOX9 near the HMG-box at arginine residues (R74 and R152) is also observed in chondrocytes, driving cell cycle progression [89].

**Figure 4:**
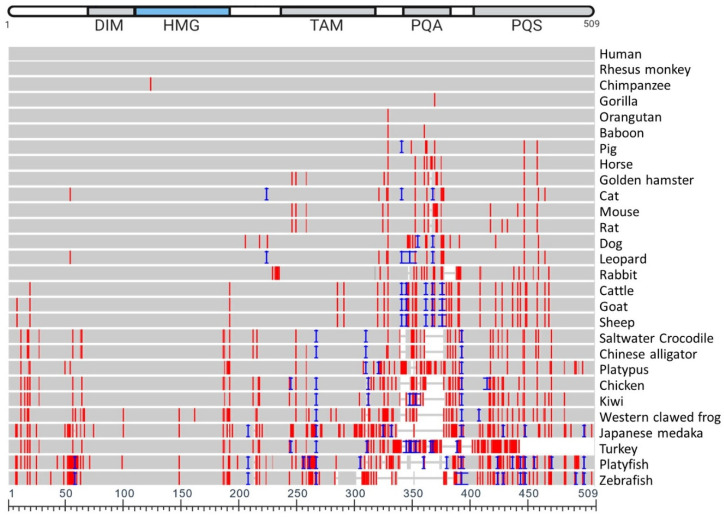
Multiple sequence alignment of SOX9 amino acid sequences across 28 species, compared to human SOX9 reference. Human SOX9 protein along the top of the multiple sequence alignment, indicating the dimerization domain (DIM), DNA-binding HMG-box, transactivating domain in the middle of the protein (TAM), the proline, glutamine, and alanine rich region (PQA) and the proline, glutamine, and serine rich region (PQS). In the multiple sequence alignment, grey indicates identical sequence to human SOX9 at each residue, red indicates different residue, and blue indicates an insertion. Images created through NCBI COBALT (Constraint-based multiple alignment tool) with 28 sequences selected through the NCBI Orthologues feature, NCBI Multiple Alignment Sequence Alignment Viewer, Version 1.19.1 (https://www.ncbi.nlm.nih.gov/gene/6662 (accessed on 16 February 2021)).

SOX9 protein sequences from 28 vertebrates were aligned (Figure 4). The multiple sequence alignment (Figure 4) highlights the high degree to which the amino acid sequence is conserved across all 28 species examined, and Table 1 lists the identity concordance, ranging from 100% between human and rhesus monkey, through to the lowest level of 70.34% with the zebrafish (58 mismatches, 47 residue difference in length). This table also indicates the specific number of residues different between various species and human SOX9. 

**Figure 5:**
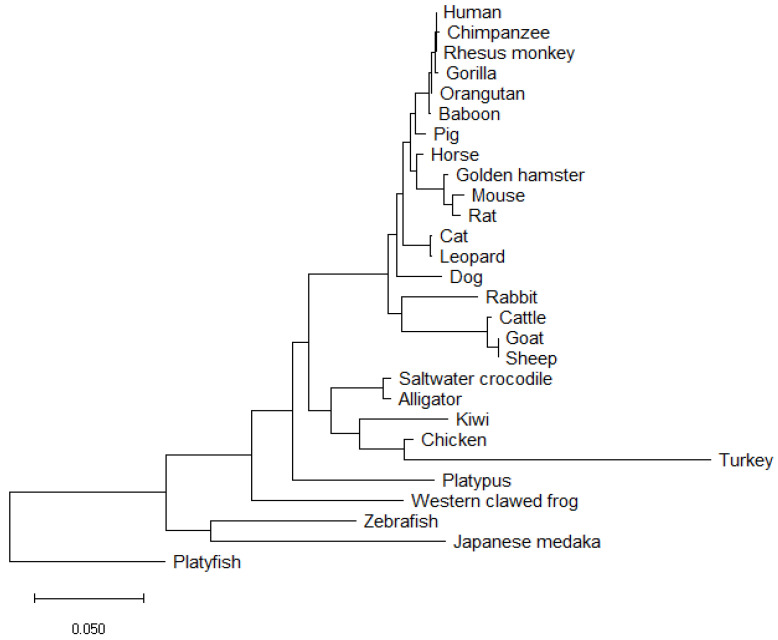
The evolutionary history of SOX9 was inferred using the Neighbour-Joining method [98]. The optimal tree is shown. The tree is drawn to scale, with branch lengths in the units of the number of amino acid substitutions per site to infer evolutionary distance, computed using the Poisson correction method [99]. This analysis involved the amino acid sequences of the SOX9 protein from 28 species aligned using NCBI COBALT [90]. All ambiguous positions were removed for each sequence pair (pairwise deletion option). Phylogenetic tree generated using MEGA X software [100].

SOX9 proteins vary in amino acid length across vertebrate species as shown in Table 1; for example, from 462 amino acids in the zebrafish to 529 in the leopard. It is apparent in the multiple sequence alignment (Figure 4) that there are very little differences in the HMG box across the 28 species examined. Little difference also exists in the DIM, and variations in sequence are most abundant towards the C-terminus. Amino acid conservation of SOX9 relative to human varies from 99.61% (baboon) to 100% (rhesus monkey) concordance in primates. In rodents, this varies from 96.27% (rat) to 96.86% (hamster). Of the avian species included, the kiwi and the chicken show 82.73% and 83.52% concordance, respectively. However, the turkey shows a low level of concordance to human (71.64%); as observed in the phylogenetic tree (Figure 5), the turkey SOX9 sequence also varies to that of the phylogenetically related chicken (as evident from the extended branch). The high variation in the C-terminus region in these vertebrate species, specifically in the PQA and PQS transactivating domains, indicate that the transcriptional activation functions have evolved.

In zebrafish (*Danio rerio*), Japanese medaka (*Oryzias latipes*) and platyfish (*Xiphosphorus maculatus*), gene duplication has resulted in the occurrence of two orthologs: *sox9a* and *sox9b* [91]. Through analysis using the dN/dS ratio, a measure of evolutionary pressure on protein-coding regions, it becomes apparent that the *sox9* paralogs have similar coding sequence divergence and higher dN/dS ratio than non-teleost orthologs—indicating that there may have been relaxed negative selection on both *sox9a* and *sox9b* after gene duplication [91]. Retention of the two copies may occur as a result of advantageous mutations leading to new functions. This may explain why *sox9a* and *sox9b* are both expressed in the eyes and brain of zebrafish, Japanese medaka, and platyfish, yet *sox9a* is expressed in the testis of only the zebrafish; and why in the Japanese medaka and the playtfish, *sox9a* is expressed in the ovary [91]. Conversely, in zebrafish *sox9b* is expressed in the ovary, whereas in Medaka *sox9b* is initially expressed in the gonads of both sexes, but later becomes testis-specific at the time of testicular tubule development [91,92,93,94].

Comparing SOX9 orthologs indicates that while in humans the PQA comprises a 35–45 amino acid region rich in proline (42%), glutamine (39%) and alanines (18%); in lower vertebrates it has only 5–14 residues, with only a few glutamines in ancient fish [83]. Comparison of amino acid sequences of SOX9 from various vertebrate species highlights the high degree that the entire sequence is conserved across species (Figure 4), particularly around the HMG-box and near the N-terminus. The sequence similarity depicted in Figure 3 indicates the lower degree to which fish (Japanese medaka, platyfish and zebrafish) SOX9 sequences are conserved against human SOX9, especially near the C-terminus. The regions that are more highly conserved (near the N-terminus) are also more commonly associated with regulation during chondrogenesis, implying that the SOX9 transcriptional function during chondrogenesis is more highly conserved than during sex determination.

The neighbour-joining phylogenetic tree (Figure 5) indicates high conservation of SOX9 between supraprimate species, rodents, reptiles and birds. The mechanism of sex determination varies between these species, yet the degree to which SOX9 protein sequences vary appears minor. Monotreme species, such as the platypus, present an interesting evolutionary link between sex determination pathways. One of the five platypus X chromosomes confers homology to the avian Z chromosome [10,11]. In platypus, reverse gene dosage compared to birds is observed for *DMRT1*, the avian Z-linked sex gene. Male birds (ZZ) express higher levels of DMRT1, whereas in the platypus *Dmrt1* has been mapped to the X_5_ chromosome, of which females have two [10,95]. While the platypus SOX9 protein is orthologous to that of other species, it has been ruled out as the sex determining switch [24].

## 6. Conserved Function of Vertebrate SOX9 Protein

A pair of XY chromosomes with SRY initiating the gene cascade for male sex determination evolved between 166 and 148 million years ago, and has remained stable in most mammals [96]. *SOX9*, a target of SRY, is arguably the most critical gene in the sex determination cascade of many vertebrates since it is highly conserved across species. However, the role of *SOX9* in the male sex determination cascade across vertebrate species is not as conserved as to be expected. The understanding of the fundamental role that SOX9 plays in male sex determination has continued to expand since 1996, when the sexually dimorphic expression of *Sox9/SOX9* became evident in mouse and chicken embryos [97] —two phylogenetically distant species, with different sex determination switch mechanisms. Expression of SOX9 in the genital ridge driving Sertoli cell differentiation is observed in mouse, chicken, turtle, [38], as well as a similar expression pattern of SOX9 in chicken and mouse skeletal systems [97]. Thus, the role that SOX9 plays within vertebrates appears to be pivotal, regardless of the sex determining switch or downstream mechanism.

*SOX9* is a central ‘hub’ gene of gonadal development, with a conserved role in testis development across many vertebrates—including at the time of sex determination, and shortly after during gonad differentiation. In mammals, SOX9 is expressed in pre-Sertoli cells at the time of male sex determination and in mammals, SOX9 is both necessary and sufficient for testis development. In both mice and humans, loss of SOX9 in XY gonads leads to male-to-female sex reversal and ectopic expression of SOX9 in XX gonads results in testis development [101,102,103,104], highlighting the essential and central role of *SOX9* as a ‘hub’ gene in male sex determination. 

The Sox9 signaling pathway, as observed in mammalian testis development, also induces ovary-testis transition in zebrafish [105]. Similar to mammals, the relevant paralog of *sox9* for this transition, *sox9a*, is expressed in the undifferentiated testis before the onset of *Amh* expression [92]. In addition, suppression of the *sox9b (sox9a2)* paralog by knockdown of the male switch *Dmy* in the Japanese rice fish *Oryzias latipes* (Medaka) promotes the female cascade, resulting in fertile male-to-female sex reversal [106].

Avian sex determination mechanisms have yet to be fully explored, but *DMRT1* has been shown to hold a key role in male sex determination [8]. In the developing undifferentiated chicken testis, SOX9 is expressed after DMRT1 from E5.5, and overexpression of *DMRT1* in E7.5 female chicken gonads induces localised activation of *SOX9*, resulting in the development of cord-like structures in masculinized gonads [107].

In contrast to the vertebrate species above, analyses of SOX9 expression in alligator and turtles indicate that SOX9 has an important role in testis differentiation, rather than sex determination, in reptiles with temperature-dependent sex determination (TSD) and reptiles with chromosomal sex determination (GSD) mechanisms, In *Alligator mississippiensis*, *SOX9* is expressed in the embryonic testis during structural organisation at the end of the temperature-sensitive period [5]. The sea turtle *Lepidochelys olivacea* expresses *SOX9* in both male and female gonads until the critical sex determining thermosensitive stage 24, from which *SOX9* is maintained in differentiating male gonads at male-promoting temperatures, and downregulated in female gonads within two days as a result of female-promoting temperatures [108,109]. Both *Dmrt1* and *Sox9* mRNA expression increases in the snapping turtle *Chelydra serpentina* at male-promoting temperatures, indicating both are part of a core testis-determining mechanisms [110]. Chromosomal sex-determined reptiles such as the Chinese soft-shelled turtle *Pelodiscus sinesis* (ZZ/ZW) also have a complex network for sex determination. *Sox9* expression in this species is first detected during early gonad differentiation. In contrast, *Dmrt1* expression precedes male gonad differentiation, and ectopic expression of *Dmrt1* can up-regulate *Sox9* to induce masculinisation in ZW females [111]. Interestingly, *Amh* expression also precedes male gonadal differentiation in *P. sinesis*, and overexpression of *Amh* leads to ectopic activation and expression of *Sox9*, and female-to-male sex reversal [112].

Studies in amphibians have shown that the spatiotemporal expression of SOX9 in the gonads can differ quite significantly between closely related species. Like zebrafish and Medaka, the frog *Xenopus laevis* has two *Sox9* paralogs (*Sox9a* and *Sox9b*), and both are significantly upregulated in the testes [113]. Both paralogs are expressed early in the undifferentiated developing testes, suggesting a role in male sex determination. This is surprisingly different to the expression pattern of SOX9 observed in *Xenopus tropicalis*; in both sexes, *SOX9* is upregulated only after the gonads have differentiated. In the testis, SOX9 is restricted to the nucleus of Sertoli-like cells similar to that in other vertebrates [114]. However, in the ovary, SOX9 is first localized to the cytoplasm of previtellogenic oocytes then localized to the nucleus of vitellogenic oocytes [114]. These data indicate the crucial role that SOX9 orthologs play in a variety of stages in a tissue-specific manner during embryogenesis, with functions extending beyond that of just testicular development.

*Sox9* expression is up-regulated in a male-specific manner for testes development to occur in many vertebrate species, such as chicken, mouse and alligator [115]. The influence of SOX9 on Sertoli cell differentiation appears highly conserved across species, yet the role *SOX9* plays within the male sex determination gene cascade varies, implying varied regulatory pathways. *SOX9* is observed in a male-specific, testis-specific manner for alligator testis differentiation, with an unknown regulator controlled via environmental temperature [5,116]. In chickens, expression of *AMH* is detected earlier than that of *SOX9*, despite SOX9 initiating *Amh/AMH* expression in mouse and human [117]. This suggests that the genes and processes regulated by SOX9 in gonadal development are not strictly conserved across species. RNA-seq and ChIP-Seq of mouse and chicken developing chondrocytes and Sertoli cells indicates that of the SOX9 target genes examined, there was high similarity in chondrocytes but not in Sertoli cells [118]. This indicates that chicken and mice, two vertebrates that are not phylogenetically close, harbour cell-specific binding preferences of SOX9, and that the regulatory targets of SOX9 in testis development differ between the species. Recently, the evolutionary plasticity of the gonad was further highlighted that essential supporting cell lineages in chickens are not derived from the coelomic epithelium such as in mice, but from mesenchymal origin [119]. This shows that there are fundamental differences between chicken and mouse gonad development. Surprisingly, other XY/XX chromosomal species such as horses, cattle, dogs, and cats have been reported to experience XY Sex Reversal, the mechanism by which this occurs is much less understood [120].

Given that *SOX9* has a critical role in the sex determination cascade of many vertebrates and is the ‘hub’ gene in mammalian gonadal development, it might seem surprising that *SOX9* did not evolve as the key switch in any of the vertebrate species examined to date. This could be due to the fact that *SOX9* has vital roles across in many other developmental processes. The translocation of *SOX9* to a sex chromosome may be problematic for complete organogenesis or chondrogenesis if gene dosage were altered; for example, 50%-reduced gene dosage in mice leads to bone and endocrine-specific defects similar to those observed in human haploinsufficiency syndrome Campomelic Dysplasia patients [121,122,123,124]. Thus, gonadogenesis truly highlights great cellular complexity: not only can the genetic (or environmental) trigger differ among vertebrate species, the resulting cellular assembly can too, yet resulting in such similar reproductive structures.

## 7. Disorders Arising from *SOX9* Mutations in Humans 

Abnormalities in sex differentiation and gonadogenesis can result in Disorders of Sex Development (DSD). This term encompasses a spectrum of disorders in which chromosomal, gonadal or anatomical sex is atypical [125]. The rate of congenital DSDs is estimated at 1:200 [126]. DSDs are categorised into three types on the basis of sex chromosome content: 46,XY DSD, 46,XX DSD, and sex chromosome DSDs; the most common being Klinefelter syndrome (XXY-XXXY aneuploidy) and Turner syndrome (XO aneuploidy) [127]. Mutations to *SRY* account for approximately 15% of 46,XY DSD [128]; while mutations in other genes such as *NR5A1* or *SOX9* are known to cause 46,XY DSD as part of a syndrome. To date, a genetic diagnosis is not achieved in around 50% of XY DSD cases [128,129]. Genetic diagnosis of ambiguous genitalia is particularly challenging and a clinical algorithm can facilitate this [130].

Mutations can occur within the *SOX9* coding region itself or in the non-coding regulatory region. Mutations within the gene may affect protein function, resulting in loss-of-function (complete or partial); gain-of-function; or dominant-negative mutations. If a mutation occurs in the non-coding regulatory sequences of genes, this does not impact the protein sequence but may instead affect expression of the gene in a specific tissue or a specific enhancer or repressor.

**Figure 6:**
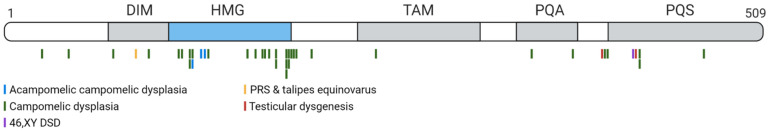
Human SOX9 protein, indicating the dimerization domain (DIM), DNA-binding HMG-box, transactivating domain in the middle of the protein (TAM), the proline, glutamine, and alanine rich region (PQA) and the proline, glutamine, and serine rich region (PQS). Dashes underneath indicate missense single base-pair mutations causing a triplet change and subsequent amino acid change, causing various phenotypes [82,101,128,131,134,135,136,137,138,139,140,141,142,143,144,145,146,147,148,149,150,151,152,153,154,155,156].

Heterozygous loss-of-function mutations occur within either the coding or regulatory region for *SOX9* in patients with Campomelic Dysplasia (CMPD; OMIM 114290). CMPD is a severe and fatal skeletal malformation syndrome in which 70% of 46,XY patients have either ambiguous genitalia or develop as females due to 46,XY gonadal dysgenesis [131]. Typical skeletal features of CMPD patients with mutations in the *SOX9* coding region include bowed lower limbs, hypoplastic scapulae, narrow iliac wings, and non-mineralised thoracic pedicles [132]. An atypical form of CMPD, known as acampomelic campomelic dysplasia occurs as a result of alterations between 50–375 kb upstream of *SOX9* resulting in a phenotype similar to that of CMPD but with the absence of bowed limbs [133]. In addition to these phenotypes in the developing bones and gonads, patients with CMPD may also show defects within other tissues in which *SOX9* is expressed, such as brain (e.g., the absence of olfactory bulbs), heart, kidney and lung abnormalities [132]. Loss-of-function mutations within the coding sequence of *SOX9* occur, for example, in the DNA-binding domain HMG-box, the nuclear localisation signals (NLS), or in the transactivating domain (Figure 6) [82,101,128,131,134,135,136,137,138,139,140,141,142,143,144,145,146,147,148,149,150,151,152,153,154,155,156]. In some CMPD patients with associated sex reversal, the *SOX9* coding sequence is not affected, but translocation breakpoints have been identified in the *SOX9* regulatory region up to several hundred kb upstream of *SOX9*.

As well as *SOX9* being responsible for the syndrome Campomelic Dysplasia with associated 46,XY DSD, *SOX9* mutations can also cause isolated DSDs including 46,XX testicular DSD (OMIM: 278850, 300833, and 400045), which involves the development of histologically normal testis in 46,XX individuals; and 46,XX ovotesticular DSD (OMIM: 400045) which involves the development of ovotestis in which both ovarian and testicular tissue is present. 46,XY partial testicular dysgenesis (OMIM: 154230, 300018, 612965, 613762, 615542, 616067, and 616425) can result in ambiguous genitalia varying along a spectrum from almost female phenotype, to an almost male phenotype [157,158]. 46,XY DSD can be caused by heterozygous deletions at the *SOX9* locus, removing parts of the upstream *SOX9* regulatory region [48]. 46,XX testicular/ovotesticular DSDs can occur in an *SRY*-independent manner as indicated in a study in which only six of 17 patients with 46,XX ovotesticular/testicular DSD were *SRY*-positive [159]. Genomic duplications involving *SOX9* can be the causative mutation, likely resulting in activation of *SOX9* expression in the XX gonad. Three patients with total gene duplication of SOX9 [103,160,161], 17 patients with duplication of the upstream regulatory region [48,63,64,162,163,164,165,166,167], and one patient with a triplication of the regulatory region [65] have been previously identified. *Sox9* knock-out mice show sex reversal [102] and overexpression of *Sox9* in XX mice induced male development [168]; thus the same ‘disorders’ or developmental differences can be induced in mice as seen in humans. Furthermore, heterozygous deletion of *Sox9* in mouse mimics the sex reversed phenotype as seen in CMPD in humans [169].

Recently, the first gain-of-function missense *SOX9* variant (p.Glu50Lys) was identified in a patient with 46,XX ovotesticular DSD [170]; suggesting that mutations in the *SOX9* gene can result in both loss- or gain-of-function. In vitro experiments showed that the SOX9 variant increased transactivation of an mTESCO-luc reporter when compared to wildtype SOX9, whereas female mice carrying this SOX9 variant did not show abnormalities of external or internal genitalia. However, it is not unusual to experience discordance in DSD-associated gene expressivity between humans and mice [170]. *SOX9* mutations identified in DSDs are often involved in human infertility caused by testis gonadal dysgenesis, or XY and XX sex reversal. In a family with two 46, XX infertile males, both have a 96 kb triplication 500 kb upstream of *SOX9* and present with hypotrophic testes containing no sperm [165].

SOX9 might also be involved in human hair follicle development, similar to its role in mice [171]. Congenital hypertrichosis is a rare condition characterized by excessive hair growth in humans. In a family with hypertrichosis, a large 2.4 Mb duplication 975 kb upstream of *SOX9* was identified and dramatically reduced the expression of *SOX9* in hair follicles [172].

Mutations in *SOX9* enhancers upstream of *SOX9* can also be associated with isolated craniofacial anomalies of the CMPD syndrome called Pierre Robin sequence (PRS; OMIM: 261800) [54]. This congenital syndrome is characterized by underdevelopment of the lower jaw (micrognathia), which can lead to secondary phenotypes including obstruction of the airway and retraction of the tongue. While mutations in this region do not contribute to DSDs, it is important to note the obvious presence of tissue-specific enhancers of *SOX9* presenting an excellent opportunity to further investigate long-range regulation of genes crucial for development. Misdiagnosis and mismanagement of disorders due to a lack of genetic information associated with development of the testis can induce psychological and physiological risks, including gonadoblastomas, subfertility, gender dysphoria, anxiety, depression and reduced psychosexual wellbeing [173,174,175], making it ever-more-important that understanding of DSDs pathogenesis continues. 

## 8. Gonad Plasticity: The Role of SOX9 in Transdifferentiation

The question of whether “terminally” differentiated cells, such as Sertoli cells and granulosa cells, can switch from one fate to the other was first posited in 1988, when it was suggested that an ovary-determining signal produced by an XX component may pre-empt the testis-determining action of the Y chromosome [176]. Two decades later, Uhlenhaut et al. [14] indeed demonstrated transdifferentiation of adult mouse ovaries to testes through an inducible deletion of *Foxl2*, a pro-ovarian gene; and in the reverse, Matson et al. [15] showed transdifferentiation of adult testes to ovaries via loss of *Dmrt1.*

From observations of the polled intersex syndrome (PIS) in XX female-to-male -sex reversed goats which contain a 11.7 kb deletion of the *FOXL2*-containing region on chromosome 1, Uhlenhaut et al. provided evidence supporting the theory that maintenance of the male fate of the gonad is a lifelong, active process, counter to the previous idea of terminal differentiation and permanent cell fate. Through inducible deletion of *Foxl2* in adult mouse ovaries, upregulation of male-specific markers such as SOX9 was detected, with granulosa cells undergoing transdifferentiation to appear as testicular Sertoli cells (including the tripartite nuclei and cytoplasmic extensions). Histological analysis showed that three weeks after *Foxl2* deletion, the ovarian follicles took on the appearance of testicular seminiferous tubules, with granulosa cells and theca cells transdifferentiating into Sertoli-like and Leydig-like cell lineages, respectively. Molecular studies unveiled that in sex-reversed gonads, the deletion of *Foxl2* led to not only notable expression of *Sox9*, but also *Dax1*, *Dhh* and *Dmrt1*. The rapid upregulation of *Sox9* indicates a direct transcriptional repression of *Sox9* by FOXL2 in the ovary, possibly achieved via the testes-specific enhancer TESCO [58]. Indeed, ChIP assays confirmed that FOXL2 directly bound to *Tesco* in vivo, and that specific deletion of *Foxl2* led to strong *TESCO-ECFP* activation within the transdifferentiating follicles. Furthermore, in vitro FOXL2 can attenuate TESCO activation by SF1, SRY/SF1, or SOX9/SF1. Additionally, in vitro results showed that FOXL2 synergised with ESR-1 (Estrogen Receptor 1) to repress the TESCO element [14]. Further elucidation by Georges et al. indicate that repression of *SOX9* via FOXL2 occurs via multiple pathways, more so through ESR2/E2 and independent of estrogen, not via binding of FOXL2 or ESR1 through the TESCO enhancer [177]. The theory that maintenance of sexual fate requires the repression of genes of the opposite sex was further supported by Matson et al., where the converse male-to-female transdifferentiation highlights the role in which *Dmrt1* is essential for maintenance of mammalian testis differentiation [15] and the two-way plasticity of gonadal development. Sertoli-cell specific loss of the DMRT1 protein in mice induced *Foxl2* expression, with the antagonistic relationship between pro-ovarian *Foxl2* and pro-testes *Dmrt1* proving the labile fate of gonadal cells. One month after deletion of *Dmrt1*, adult XY males had morphologically appearing Sertoli cells (tripartite nuclei) expressing both SOX9 and DMRT1, right next to morphologically appearing granulosa cells expressing only FOXL2, with the entire testicular tissue restructuring to appear more similar to typical ovarian morphology. Theca cells developed, and mRNA for oestrogen precursors HSD17β1 and CYP19A1/aromatase were detected in mutant gonads. Oestrogen signalling acts in conjunction with FOXL2 to repress *Sox9* transcription. This indicates that the antagonistic relationship persists into adulthood, with the supporting cells also remaining labile after differentiation.

In addition to *Foxl2*, targeted deletion of *Wnt4* or *R-Spondin1* in mice individually results in the partial masculinisation of the embryonic XX gonad, i.e., not complete testes development [178,179]. Testis development can be induced in embryonic XX gonads lacking both *Foxl2* and *Wnt4*, due to the subsequent activation of *Sox9* which leads to the development of seminiferous tubules and spermatogenesis [180]. These findings may explain the mechanisms behind the sex-reversal seen in goats with PIS. Since *Foxl2/Wnt4* double mutant mice demonstrate that granulosa cells acquire Sertoli-like characteristics, like upregulation of *Sox9*, *Dmrt1*, and other testis genes, Wnt signaling is important for ovarian development. Both WNT4 and RSPO1 stabilize β-catenin, and ectopic expression of its stable form in XY gonads can result in male-to-female sex reversal [181]. The Wnt/β-catenin pathway blocks testicular differentiation by repressing the expression of *SOX9*, possibly by activation of β-catenin preventing SF1 binding to the *Sox9* enhancer TESCO [181] and likely other enhancers [67].

*Sox9* regulation clearly has a crucial influence in both transdifferentiation pathways. Barrionuevo et al. investigated the effect of Sertoli cell specific *Sox9* ablation on a *Sox^−/−^* adult testis from postnatal day 60 mouse [182]. *Sox8* and *Sox9* double knockout within Sertoli cells induced testis-to-ovary reprogramming, with Sertoli to granulosa transdifferentiation as a result of *Dmrt1* downregulation [182]. *Sox8* and *Sox9* maintain basal lamina integrity to prevent testis cord disintegration and both SOX8 and SOX9 actively suppress the ovarian program during testis development [183].

**Figure 7:**
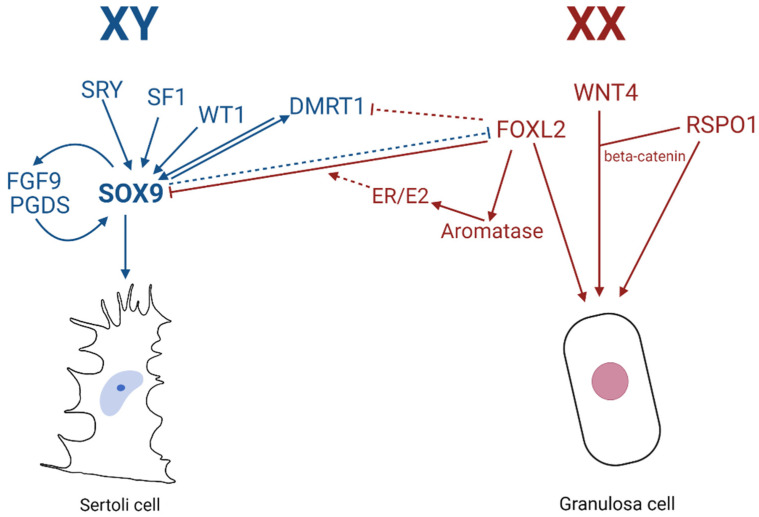
Molecular mechanisms of mammalian sex determination leading to male or female supporting cell fate.

The battle between pro-ovarian and pro-testicular influence for sex determination is evident, with gonadal suppression of *Sox9* essential for ovarian development in females, and the absence of FOXL2 (gonadal presence of SOX9) crucial for testicular development (Figure 7). These studies highlight the way in which development is not “final”, in the sense that the organs remain plastic and the constant struggle between the two competing pathways can be manipulated in favour of one over the other, regardless of chromosomal sex.

## 9. Conclusions

While the early gonad is bipotential with the capacity to develop down either sex differentiation lineage, our understanding of molecular mechanisms that drive testes differentiation is expanding through the identification of conserved elements across species, and through genome analysis of patients with DSDs. Furthermore, abnormalities arising from mutations in the *SOX9* regulatory or coding regions indicate the importance of functional SOX9 protein; craniofacial disorders, testicular dysgenesis and infertility can all arise from such mutations. *SOX9* is multifunctional, with tissue-specific regulation and roles during embryogenesis, but the plasticity of the regulation of such a conserved transcription factor demonstrates the important role *SOX9* plays to mediate both male or female cell fate. Despite the various signals acting upon the *SOX9* regulatory region—be it through a ZZ/ZW or XY/XX chromosomal system, or perhaps through temperature sex determination—the common effect of the signals seems to result in upregulation of *SOX9* to promote testicular development. Contrasting to the diversity of the regulatory region, the encoded protein is highly similar in vertebrates suggesting common downstream target genes required for testis development. SOX9 mediation of Sertoli cell development can control the downstream cellular differentiation of the bipotential germ cells and supporting cells. Furthermore, the reversal of terminal differentiation of ovarian or testicular supporting cells indicates that cell fate is not canalized, raising the possibility of unappreciated postnatal changes in human DSD.

## Figures and Tables

**Table 1:** NCBI sequences used to produce N-J phylogenetic tree and COBALT multiple sequence alignment.

NCBI Sequence ID	Amino Acid Length	Species Name	Species Common Name	Identity (%)	Coverage (%)	Mismatches
NP_000337.1	509	*Homo sapiens*	Human	-	-	
NP_001028040.1	509	*Macaca mulatta*	Rhesus monkey	100	100	0
XP_009250264.1	509	*Pan troglodytes*	Chimpanzee	99.80	100	1
XP_018883823.1	509	*Pongo abelii*	Orangutan	99.80	100	1
NP_001009029.1	509	*Gorilla gorilla gorilla*	Gorilla	99.80	100	1
XP_003913405.1	509	*Papio Anubis*	Baboon	99.61	100	2
NP_999008.2	511	*Sus scrofa*	Pig	98.04	100	8
XP_023507898.1	509	*Equus caballus*	Horse	98.04	100	10
XP_005070025.1	507	*Mesocricetus auratus*	Golden hamster	96.86	99.61	14
XP_023099583.1	511	*Felis catus*	Cat	96.68	99.80	13
NP_035578.3	507	*Mus musculus*	Mouse	96.46	99.61	16
XP_032769123.1	507	*Rattus rattus*	Rat	96.27	99.61	17
NP_001002978.1	513	*Canis lupus familiaris*	Dog	94.75	99.80	21
XP_019321937.1	529	*Panthera pardus*	Leopard	93.40	99.80	13
XP_008269985.1	497	*Oryctolagus cuniculus*	Rabbit	91.75	97.64	30
XP_024836864.1	524	*Bos taurus*	Cattle	90.84	100	33
XP_017919394.1	525	*Capra hircus*	Goat	90.48	100	34
XP_027829812.1	526	*Ovis aries*	Sheep	90.30	100	34
XP_020858282.1	513	*Phascolarctos cinereus*	Koala	89.60	98.82	38
XP_006029531.1	494	*Alligator sinesis*	Chinese alligator	88.13	96.07	36
XP_019397875.1	494	*Crocodylus porosus*	Crocodile	88.52	96.07	34
XP_001506094.2	508	*Ornithorhynchus anatinus*	Platypus	87.55	98.82	53
NP_989612.1	494	*Gallus gallus*	Chicken	83.52	94.50	45
NP_001016853.1	482	*Xenopus tropicalis*	Western clawed frog	81.64	94.11	61
NP_001098556.1	476	*Oryzias latipes*	Japanese medaka	72.74	80.94	85
XP_025923282.1	500	*Apteryx rowi*	Kiwi	82.73	94.70	46
XP_010719808.1	451	*Meleagris gallopavo*	Turkey	71.64	80.94	66
XP_005807407.1	495	*Xiphosphorus maculatus*	Platyfish	70.58	91.75	88
NP_571718.1	462	*Danio rerio*	Zebrafish	69.96	85.46	60

N.B. Percent identity calculated as the number of mismatches in alignment row relative to human SOX9, where the alignment length is the aligned sequence minus gaps. Percent coverage is calculated as the number of aligned residues in alignment row relative to the length of human SOX9. Mismatches indicate the raw number of residues different per row in comparison to human SOX9. Alignment created via NCBI Orthologues (https://www.ncbi.nlm.nih.gov/gene/6662/ (accessed on 16 February 2021)).and COBALT Multiple Alignment Tool [90].

## Data Availability

No new data were created or analyzed in this study. Data sharing is not applicable to this article.
